# Identification of hub genes in prostate cancer using robust rank aggregation and weighted gene co-expression network analysis

**DOI:** 10.18632/aging.102087

**Published:** 2019-07-15

**Authors:** Zhen-yu Song, Fan Chao, Zhiyuan Zhuo, Zhe Ma, Wenzhi Li, Gang Chen

**Affiliations:** 1 Department of Urology, Jinshan Hospital of Fudan University, Shanghai, China; 2 Department of Urology, Shanghai Ninth People’s Hospital, Shanghai Jiaotong University School of Medicine, Shanghai, China

**Keywords:** prostate cancer, hub genes, robust rank aggregation, weighted gene co-expression network analysis, bioinformatics

## Abstract

The pathogenic mechanisms of prostate cancer (PCa) remain to be defined. In this study, we utilized the Robust Rank Aggregation (RRA) method to integrate 10 eligible PCa microarray datasets from the GEO and identified a set of significant differentially expressed genes (DEGs) between tumor samples and normal, matched specimens. To explore potential associations between gene sets and PCa clinical features and to identify hub genes, we utilized WGCNA to construct gene co-expression networks incorporating the DEGs screened with the use of RRA. From the key module, we selected LMNB1, TK1, ZWINT, and RACGAP1 for validation. We found that these genes were up-regulated in PCa samples, and higher expression levels were associated with higher Gleason scores and tumor grades. Moreover, ROC and K-M plots indicated these genes had good diagnostic and prognostic value for PCa. On the other hand, methylation analyses suggested that the abnormal up-regulation of these four genes likely resulted from hypomethylation, while GSEA and GSVA for single hub gene revealed they all had a close association with proliferation of PCa cells. These findings provide new insight into PCa pathogenesis, and identify LMNB1, TK1, RACGAP1 and ZWINT as candidate biomarkers for diagnosis and prognosis of PCa.

## INTRODUCTION

Prostate cancer (PCa) is the second most frequent cancer and the fifth leading cause of death in males worldwide [1]. Nowadays, prostate specific antigen (PSA) is the only circulating biomarker routinely used for early diagnosis of PCa [2]. However, PSA screening has some limitations. In some cases, prostatitis and benign prostatic hyperplasia (BPH), which frequently affect men, can also result in increments of serum PSA [3]. In addition, since the optimal PSA expression threshold for clinical samples has not been determined [4], routine PSA screening sometimes leads to over-diagnosis and over-treatment of indolent PCa [5, 6]. In the past decade, a growing number of microarray and next-generation sequencing technologies have been used to explore novel biomarkers and therapeutic targets for PCa [7]. However, small sample sizes in individual studies and use of different technological platforms create substantial inter-study variability and difficult statistical analyses. To solve this problem, integrated bioinformatics methods such as Robust Rank Aggregation (RRA) have been utilized in various cancer studies [8–10].

In the present study, 10 microarray datasets from Gene Expression Omnibus (GEO, http://www.ncbi.nlm.nih.gov/geo/) were analyzed with RRA to identify robust and stable differentially expressed genes (DEGs) between PCa tissues and matched controls [11]. These DEGs were then adopted to find key modules associated with clinical features through weighted gene co-expression network analysis (WGCNA). Gene Ontology (GO) and Kyoto Encyclopedia of Genes and Genomes (KEGG) analyses were further conducted to assess potential functions of the genes within the key module. Four seldomly reported hub genes, LMNB1, TK1, and RACGAP1, and ZWINT, were selected to validate their diagnostic and prognostic value for PCa. In addition, two online tools, DiseaseMeth 2.0 and MEXPRESS, were used to assess the methylation status of those four hub genes, while Tumor Immune Estimation Resource (TIMER), Gene Set Enrichment Analysis (GSEA), and Gene Set Variation Analysis (GSVA) were utilized to investigate potential biological functions.

## RESULTS

### Identification of robust DEGs by the RRA method

**Figure 1:**
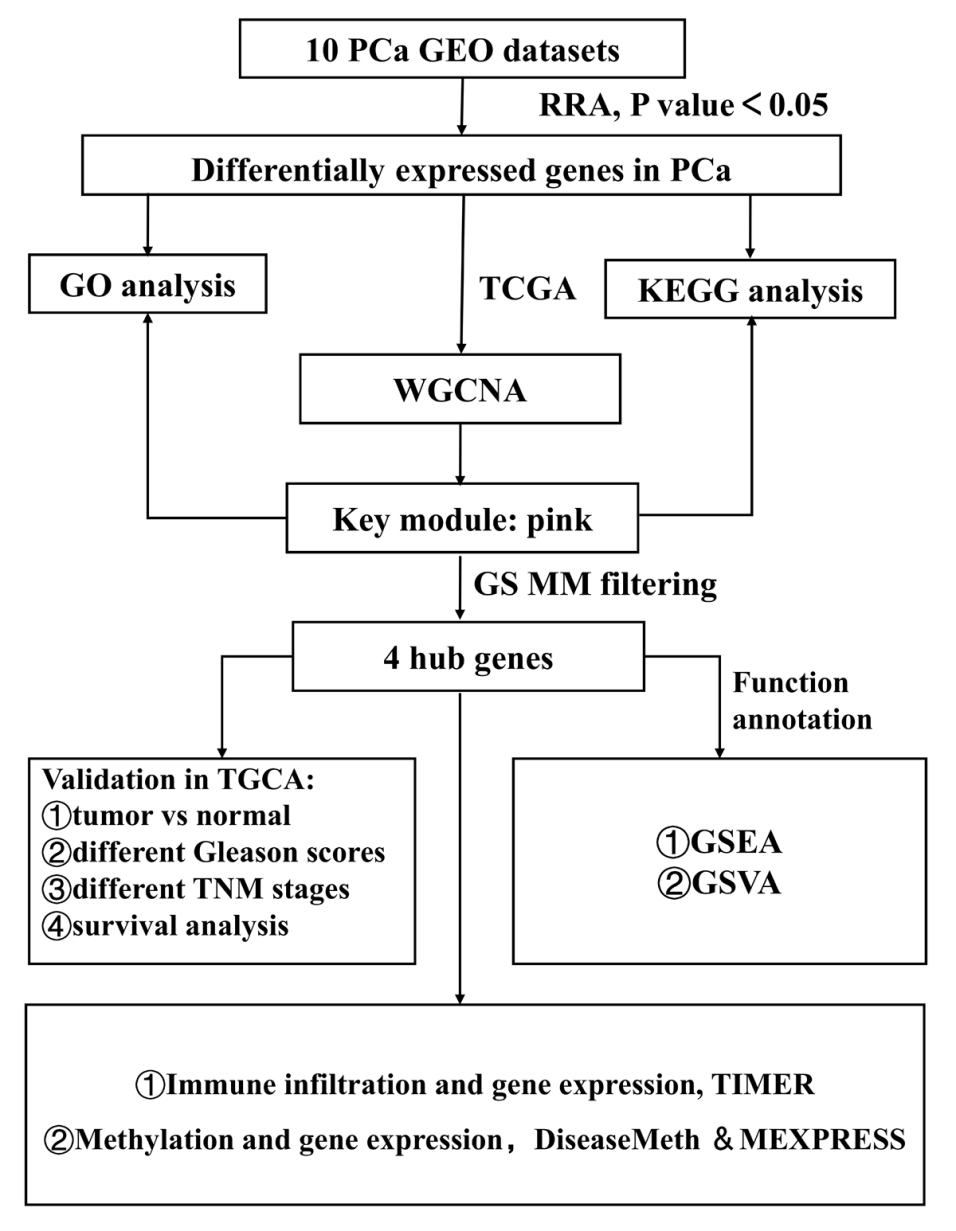
**Study workflow.** GEO, Gene Expression Omnibus; TCGA, The Cancer Genome Atlas; GO, Gene Ontology; KEGG, Kyoto Encyclopedia of Genes and Genomes; WGCNA, Weighted Gene Co-expression Network Analysis; GS, gene significance; MM, module membership; TNM, Tumor Node Metastasis; GSEA, Gene Set Enrichment Analysis; GSVA, Gene Set Variation Analysis.

**Figure 2:**
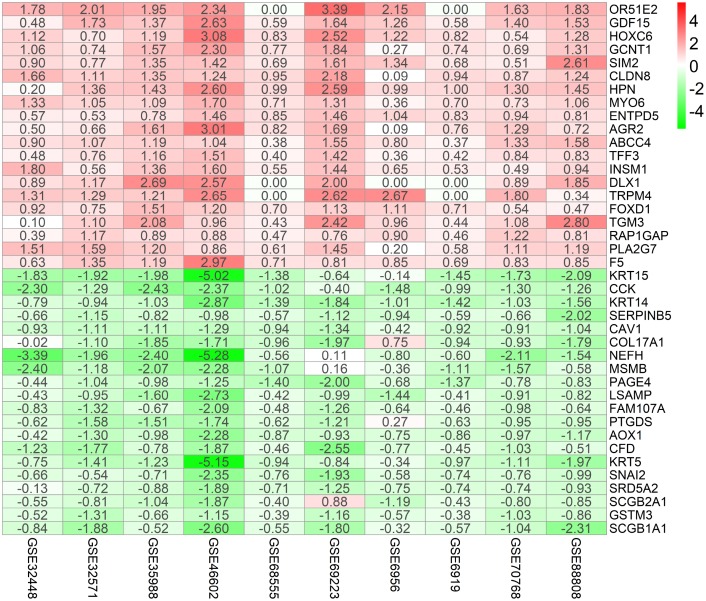
**Identification of robust DEGs by RRA analysis.** Heatmap showing the top 20 up-regulated genes and top 20 down-regulated genes according to P value. Each row represents one gene and each column indicates one dataset. Red indicates up-regulation and blue represents down-regulation. The numbers in the heatmap indicate logarithmic fold change in each dataset calculated by the “limma” R package. DEG, differentially expressed gene; GEO: Gene Expression Omnibus; RRA: robust rank aggregation.

Figure 1 shows the workflow for identification, validation, and functional analysis of DEGs. In accordance with the selection criteria, 10 eligible PCa datasets were included and used in the subsequent RRA analysis. Main characteristics of datasets, such as GEO accession ID, study country, sample information, platform ID, and number of genes for each platform, are shown in Table 1. Based on the results of RRA analysis, a total of 808 up-regulated and 930 down-regulated significant DEGs were identified (Supplementary file 1). OR51E2 was the most significant up-regulated gene (P = 4.93E-23, adjusted P = 1.94E-18), followed by GDF15 (P = 9.01E-20, adjusted P = 3.55E-15). Meanwhile, KRT15 (P = 9.07E-22, adjusted P = 3.57E-17) and CCK (P = 6.59E-20, adjusted P = 2.60E-15) were the most significant down-regulated genes in PCa samples. The top 20 up-regulated and down-regulated DEGs are shown in heatmap (Figure 2).

**Table 1:** Characteristics of the included datasets.

**Dataset ID**	**Country**	**Number of samples**	**GPL ID**	**Number of rows per platform**
GSE6919	USA	65T 63N	GPL8300	12625
GSE6956	USA	69T 18N	GPL571	22277
GSE32448	USA	40T 40N	GPL570	54675
GSE32571	Germany	59T 39N	GPL6947	48652
GSE35988	USA	59T 28N	GPL6480	41008
GSE46602	Denmark	36T 14N	GPL570	54675
GSE68555	USA	64T 63N	GPL8300	12558
GSE69223	Germany	15T 15N	GPL570	54675
GSE70768	UK	113T 73N	GPL10558	48107
GSE88808	USA	49T 49N	GPL22571	20261

Note: GSE, Gene Expression Omnibus Series; GPL, Gene Expression Omnibus Platform; T, tumor samples; N, paracancerous normal samples.

### Visualization of gene expression patterns and chromosome locations

**Figure 3:**
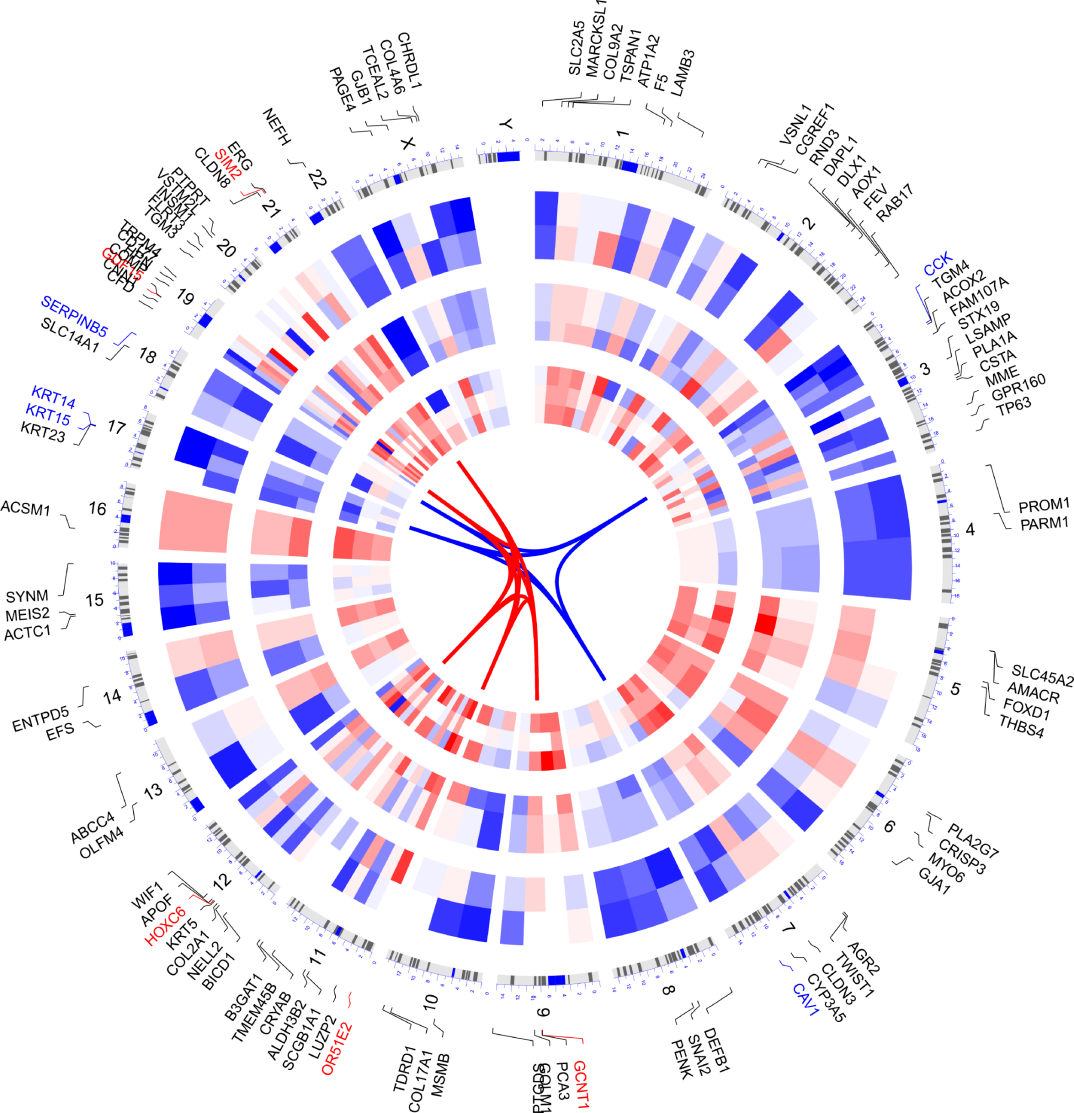
**Circular visualization of connectivity, expression patterns, and chromosomal positions of top 100 DEGs.** The 10 PCa microarray datasets from GEO are represented in the inner circular heatmaps. Red indicates gene up-regulation, blue represents downregulation, and white denotes genes not present in a given dataset. The outer circle represents chromosomes; lines coming from each gene point to their specific chromosomal locations. The top 5 up-regulated and down-regulated genes according to adjusted P are shown in red and blue and connected with red and blue lines in the center of circles.

We selected the top 100 DEGs from RRA analysis to visualize their expression patterns across the 10 datasets included in this study, as well as their chromosomal locations (Figure 3). Chromosome 3 contained most DEGs. These DEGs were distributed in all chromosomes except for chromosome Y, which showed no alterations. The top 5 upregulated genes according to adjusted P, i.e. OR51E2, GDF15, HOXC6, GCNT1, SIM2, were distributed in chromosomes 11, 19, 12, 9, and 21. The top 5 downregulated genes (KRT15, CCK, KRT14, SERPINB5, and CAV1) were located in chromosomes 17, 3, 17, 18, and 7.

### Functional enrichment analysis of DEGs

**Figure 4:**
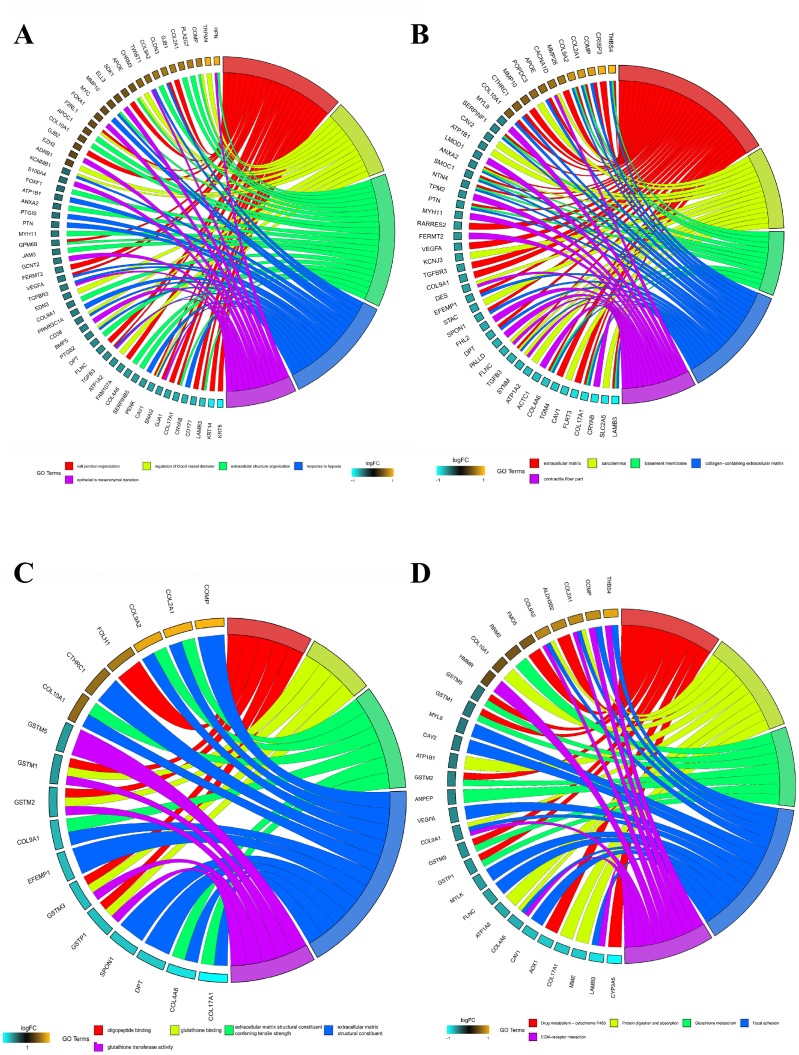
**GO and KEGG analysis of top 300 DEGs.** (**A**) Chord plot depicting the relationship between genes and GO terms of biological process. (**B**) Chord plot depicting the relationship between genes and GO terms of cellular component. (**C**) Chord plot depicting the relationship between genes and GO terms of molecular function. (**D**) Chord plot indicates the relationship between genes and KEGG pathways. GO, Gene Ontology; KEGG, Kyoto Encyclopedia of Genes and Genomes.

The top 300 DEGs were chosen to perform GO and KEGG analyses. We detected enrichment in several biological process GO terms such as cell junction organization, regulation of blood vessel diameter, extracellular structure organization, response to hypoxia, and epithelial to mesenchymal transition (Figure 4A). In terms of cellular component, extracellular matrix was the most significantly enriched GO term (Figure 4B). What’s more, some molecular component GO terms, such as glutathione binding, extracellular matrix structural constituent, were enriched (Figure 4C). As to KEGG pathway analysis, drug metabolism-cytochrome P450, protein digestion and absorption, glutathione metabolism, focal adhesion, and ECM-receptor interaction, were mostly associated with these genes (Figure 4D).

### WGCNA and identification of the key module

**Figure 5:**
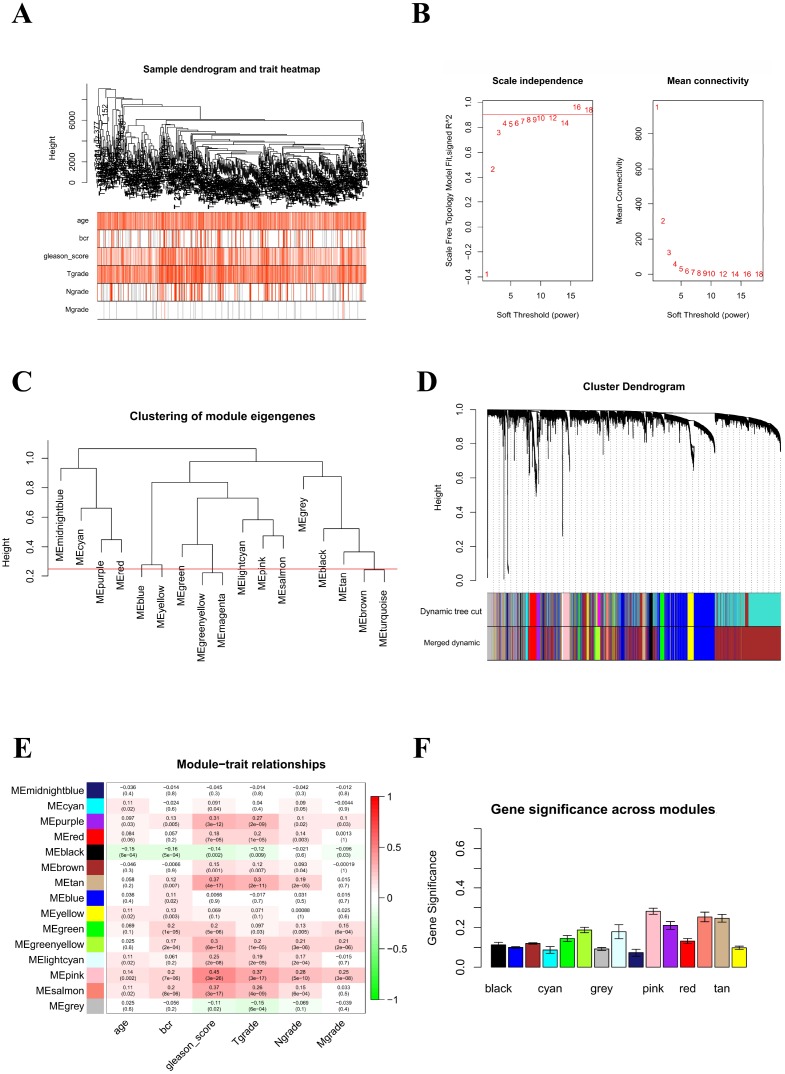
**Identification of key modules correlated with clinical traits in the TCGA-PRAD dataset through WGCNA.** (**A**) Clustering dendrograms of genes. The clustering was based on the TCGA-PRAD RNA-seq data of robust DEGs from RRA analysis. Color intensity varies positively with age, Gleason score, and pathological stage. In terms of biochemical recurrence, red means recurrence and white indicates no recurrence. (**B**) Analysis of the scale-free fit index (left) and the mean connectivity (right) for various soft-thresholding powers. (**C**) Clustering of module eigengenes. The red line indicates cut height (0.25). (**D**) Dendrogram of all DEGs clustered based on a dissimilarity measure (1-TOM). (**E**) Heatmap of the correlation between module eigengenes and clinical traits of PCa. Each cell contains the correlation coefficient and P value. (**F**) Distribution of average gene significance and errors in the modules associated with Gleason score of PCa.

**Figure 6:**
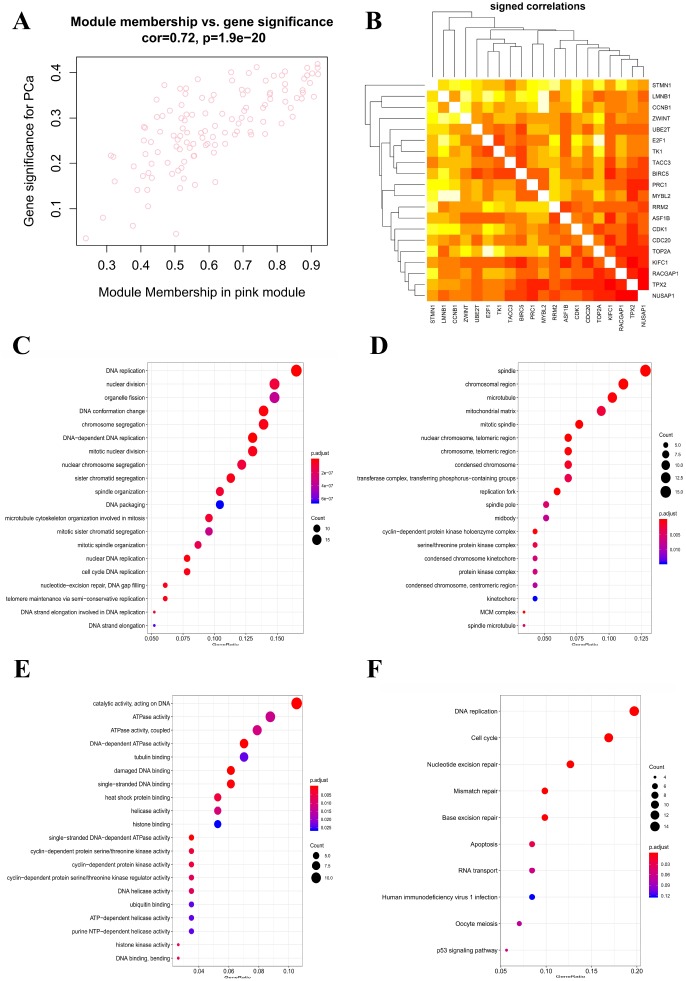
**Identification of hub genes and functional annotation of the WGCNA module highly correlated with clinical traits.** (**A**) Scatter plot of module eigengenes in the pink module. (**B**) Hub genes show strong associations with each other. (**C**) Biological process GO terms for genes in the pink module. (**D**) Cellular component GO terms for genes in the pink module. (**E**) Molecular function GO terms for genes in the pink module. (**F**) KEGG analysis for genes in the pink module. GO, Gene Ontology; KEGG, Kyoto Encyclopedia of Genes and Genomes.

To find the key modules most associated with PCa clinical traits, we performed WGCNA on the TCGA-PRAD dataset incorporating the DEGs derived from the RRA analysis (Figure 5). Clinical PCa sample information such as age, Gleason score, biochemical recurrence, and TNM grades was retrieved from TCGA (Figure 5A). By setting soft-thresholding power as 6 (scale free R^2^ = 0.85) and cut height as 0.25, we eventually identified 15 modules (Figure 5B–5D; non-clustering DEGs shown in gray). From the heatmap of module–trait correlations, we identified that the pink module was the most highly correlated with clinical traits (Figure 5E), especially the Gleason score (correlation coefficient = 0.45, P = 3E-26; Figure 5F). The pink module contained a total of 120 genes, shown in Figure 6A. By setting module membership (MM) >0.8 and gene significance (GS) >0.3, we selected 20 hub genes from the pink module: RRM2, KIFC1, TACC3, PRC1, BIRC5, CDK1, ASF1B, E2F1, RACGAP1, MYBL2, TPX2, CDC20, TOP2A, NUSAP1, UBE2T, LMNB1, CCNB1, ZWINT, STMN1, and TK1. These genes, as shown in Figure 6B, were also closely related to each other. To reveal potential biological functions of the genes within the pink module, we conducted GO and KEGG analyses. The most significant GO terms for biological process, cellular component, and molecular function, as well as KEGG pathways, were shown in Figure 6C–6F. This analysis indicated that genes within the pink module were mainly involved in DNA replication and nuclear division.

### Validation of hub genes in the TCGA-PRAD dataset.

**Figure 7:**
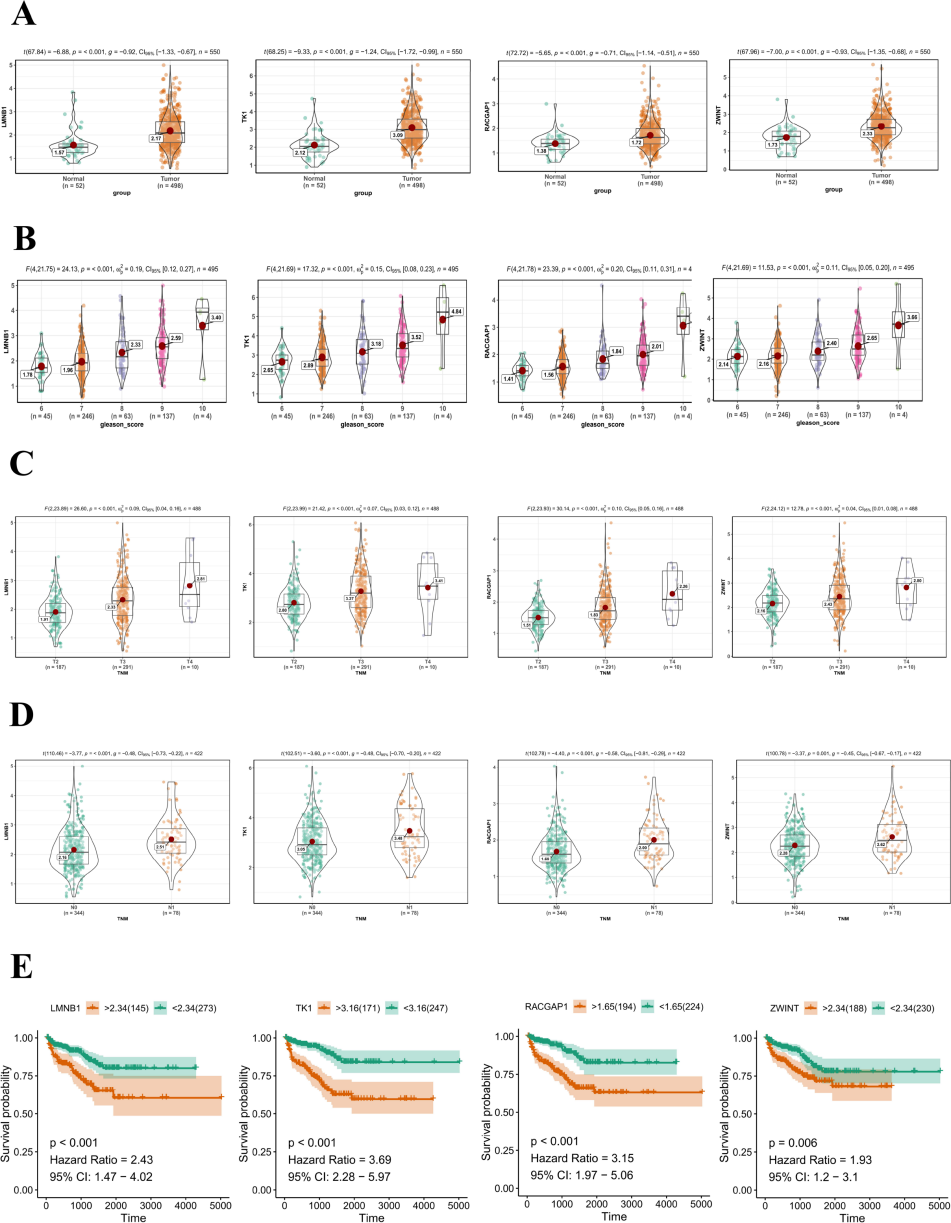
**Validation of hub genes in the TCGA-PRAD dataset.** (**A**) LMNB1, TK1, RACGAP1, and ZWINT gene expression differences between PCa and adjacent normal tissues. (**B**) Expression of LMNB1, TK1, RACGAP1, and ZWINT in PCa samples with different Gleason scores. (**C**) Expression of LMNB1, TK1, RACGAP1, and ZWINT in PCa samples with different T stages. (**D**) Expression of LMNB1, TK1, RACGAP1, and ZWINT in PCa samples with different N stages. (**E**) Association between LMNB1, TK1, RACGAP1, and ZWINT expression and disease-free survival time in the TCGA-PRAD dataset. The yellow line indicates samples with highly expressed genes (above best-separation value), and the green line designates the samples with lowly expressed genes (below best-separation value). T, Tumor; N, Node.

Among the 20 hub genes screened above, we selected four (TK1, RACGAP1, LMNB1, and ZWINT), seldomly reported before in PCa, to validate their diagnostic and prognostic value and their correlations with clinical features. It was noted that all of them were significantly up-regulated (P < 0.001) in PCa samples compared with adjacent, normal controls (Figure 7A). Furthermore, ROC curves showed their high diagnostic value as biomarkers for PCa (Supplementary Figure 1; TK1 AUC: 0.831, LMNB1 AUC: 0.786, ZWINT AUC: 0.759, RACGAP1 AUC: 0.699). In addition, LMNB1, TK1, RACGAP1, and ZWINT were significantly differentially expressed in PCa samples with different Gleason scores, T grades, and N grades, with higher expression levels indicating higher Gleason score, advanced T grades, and lymph node metastasis (Figure 7B–7D). Regarding prognosis, Kaplan-Meier curves showed that higher expression of these genes correlated significantly with poor disease-free survival (Figure 7E). Notably, all 20 hub genes in the pink module had good prognostic values (Supplementary Figure 2).

### Association between methylation and expression of hub genes

**Figure 8:**
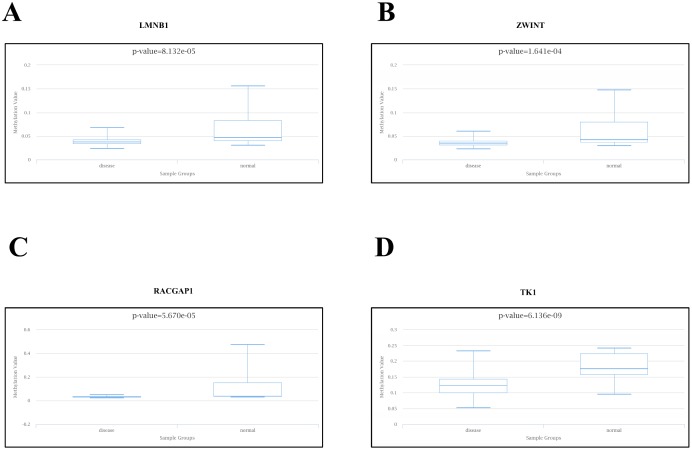
**Methylation analyses of PCa hub genes.** The methylation levels of (**A**) LMNB1, (**B**) ZWINT, (**C**) RACGAP1, and (**D**) TK1 in PCa and peri-tumoral normal tissues were examined using DiseaseMeth 2.0.

We explored the association between these four hub genes’ expression levels and their methylation status to elucidate potential mechanisms of abnormal up-regulation in PCa tissues. DiseaseMeth version 2.0 analysis showed that the mean methylation levels of LMNB1, TK1, RACGAP1, and ZWINT were all significantly lower in PCa compared with paracancerous normal tissues (Figure 8A–8D). Additionally, we found many methylation sites in the DNA sequences of LMNB1, TK1, RACGAP1, and ZWINT that were negatively associated with their expression levels using MEXPRESS (Supplementary Figure 3).

### Association of hub genes’ expression with tumor purity and immune infiltration

**Figure 9:**
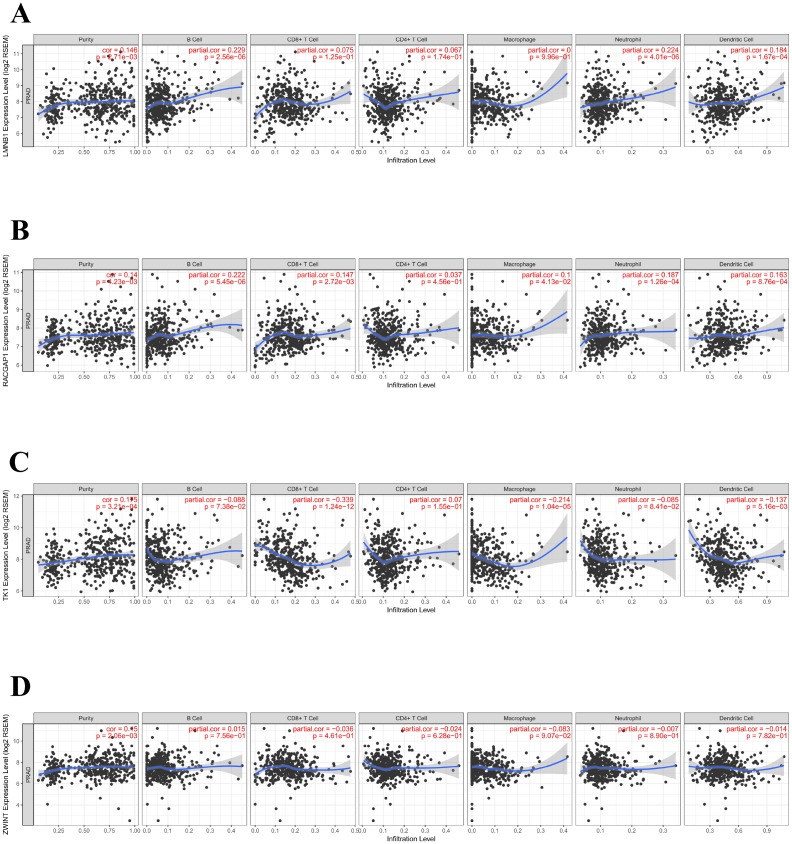
**Association of hub genes’ expression with immune infiltration in PCa**. (**A**) LMNB. (**B**) RACGAP1. (**C**) TK1. (**D**) ZWINT. P<0.05 denotes significance. Each dot represents a sample in the TCGA-PRAD dataset.

The tumor microenvironment consists of tumor cells, stromal cells, and infiltrating immune cells. We utilized TIMER to explore potential associations between the expression of PCa hub genes and both tumor purity and infiltration of immune cells. Interestingly, LMNB1, RACGAP1, TK1, and ZWINT were all positively associated with tumor purity. Conversely, no or weak associations were observed between these four genes and infiltration of B cells, CD4^+^ T cells, CD8^+^ T cells, neutrophils, macrophages, and dendritic cells (Figure 9A–9D).

### GSEA and GSVA reveal a close relationship between hub genes and tumor proliferation

**Figure 10:**
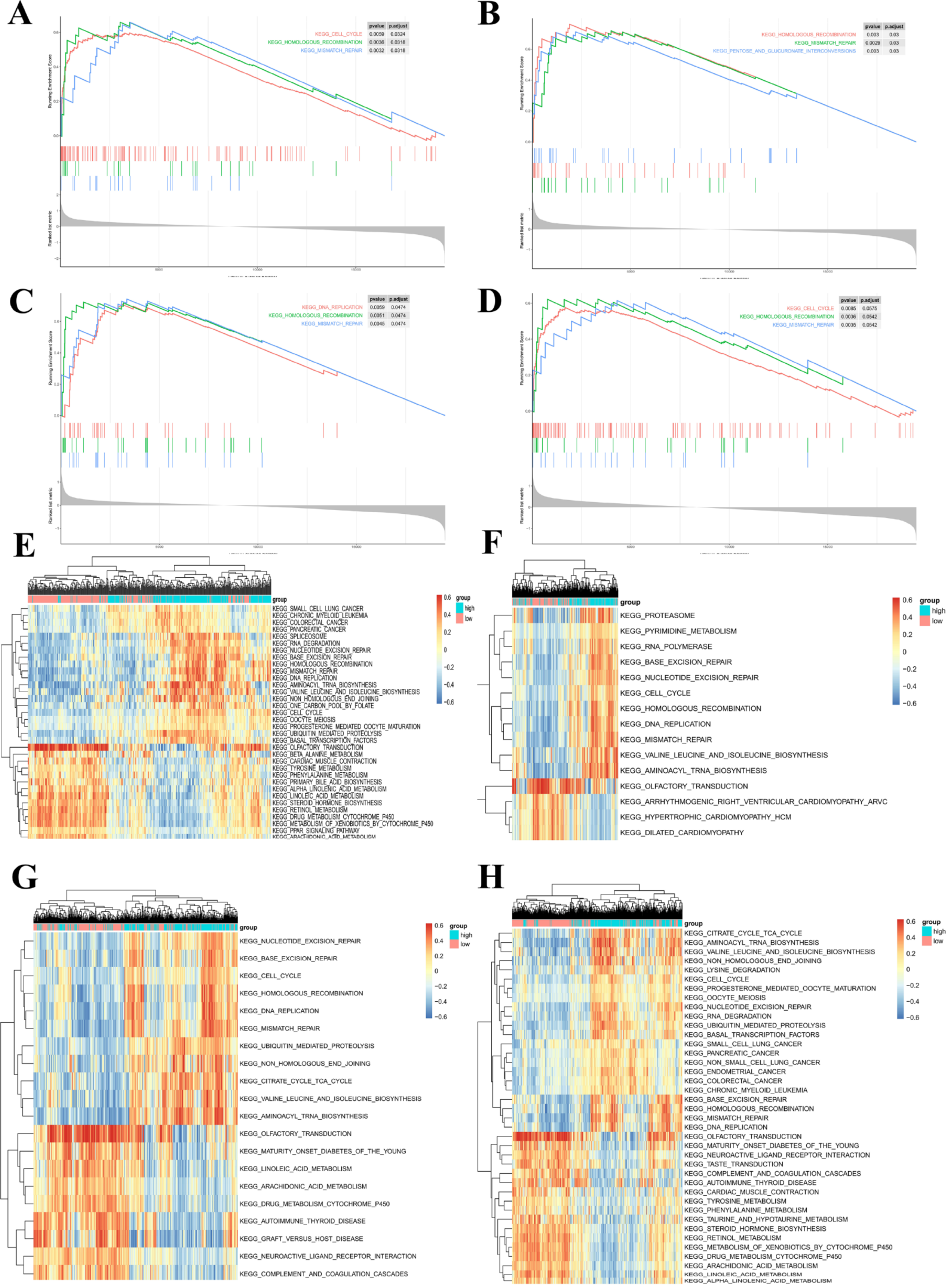
**Gene set enrichment analysis (GSEA) and gene set variation analysis (GSVA) of hub genes in the TCGA-PRAD dataset.** (**A–D**) Top 3 gene sets (according to GSEA enrichment score) enriched in the high-expression group of single hub genes. (**A**) LMNB1; (**B**) TK1; (**C**) ZWINT; (**D**) RACGAP1. (**E–H**) GSVA-derived clustering heatmaps of differentially expressed pathways for single hub genes. (**E**) LMNB1; (**F**) TK1; (**G**) ZWINT; (**H**) RACGAP1. Only signaling pathways with log(foldchange) > 0.2 are shown.

To further investigate the potential functions of LMNB1, RACGAP1, TK1, and ZWINT in PCa, we performed GSEA and GSVA on the TCGA-PRAD RNA-seq data. As shown in Figure 10A–10D, genes in high expression groups of LMNB1, RACGAP1, TK1, and ZWINT were all enriched in “Homologous recombination” and “Mismatch repair” pathways. Meanwhile, the “cell cycle” gene set was enriched in high-expression groups of LMNB1 and RACGAP1, whereas “pentose and glucuronate interconversions” and “DNA replication” were enriched in the TK1 and ZWINT high-expression groups, respectively. These gene sets with the highest enrichment scores were all closely associated with tumor proliferation. Furthermore, GSVA confirmed that these gene sets were significantly up-regulated in the high-expression groups of LMNB1, RACGAP1, TK1, and ZWINT, further suggesting their relationship with activation of proliferative processes (Figure 10E–10H).

## DISCUSSION

The pathogenesis of PCa, a complex and heterogeneous disease, remains unclear. Although numerous investigations using microarray and RNA-seq were conducted to discover novel biomarkers and therapeutic targets for PCa, inconsistencies were seen between the DEGs found in different studies [7]. To our knowledge, our work is the first to use RRA combined with WGCNA to explore novel hub genes associated with PCa. A recent study compared gene expression profiles between PCa and control samples (either para-tumoral, normal matched tissue, or normal prostate tissue from donors) for each dataset, and integrated microarray data to obtain DEGs using the RankProd method [9]. However, Chandran et al. discovered marked differences in gene expression profiles between normal prostate samples from donors and tumor-adjacent normal tissues from PCa patients [12]. To minimize variability, we only enrolled datasets containing tumor and adjacent normal samples. We integrated 10 qualified PCa datasets from GEO into the RRA method and identified many robust DEGs, some of which, such as OR51E2 [13] and GDF15 [14], have been reported to be biomarkers of PCa or play a vital role in its pathogenesis. Chromosome mapping of the top 100 DEGs showed chromosomes 3 contained most DEGs. A study reported that the androgen-driven TMPRSS2-ERG fusion was associated with deletion at chromosome 3p14, which is specific to prostate cancer [15]. Moreover, 3p13 deletion defines a subgroup of ERG+ prostate cancers characterized by aggressive clinical features and tumor recurrence [16]. All evidence suggest chromosome 3 harbor some key genes capable of influencing the pathogenesis of PCa.

Consistent with published data, the enrichment of these DEGs in several GO terms, such as cell junction organization, glutathione binding, extracellular matrix, response to hypoxia, and epithelial to mesenchymal transition, confirms their involvement in the development of PCa [17–20]. In addition, enrichment of the identified DEGs in some KEGG pathways, such as drug metabolism-cytochrome P450 and glutathione metabolism also suggests their relevance in PCa pathogenesis. Cytochrome P450 17A1 catalyzes the biosynthesis of androgens in humans [21]. Glutathione, the most abundant antioxidant in humans, plays a pivotal role in the development of cancer and drug resistance [22], and many enzymes involved in glutathione metabolism have been reported to influence the proliferation and metastasis of PCa [23, 24]. Based on the results of GO and KEGG analyses, we suggest that these DEGs are closely associated with dysregulated androgen signaling and PCa development.

Co-expression network construction and identification of hub genes through WGCNA showed that genes within the co-expression module most correlated with clinical traits of PCa samples in TCGA (pink module) were also enriched in DNA replication and nuclear division pathways through GO and KEGG analyses. After filtering for GS and MM value, we eventually obtained 20 hub genes (RRM2, KIFC1, TACC3, PRC1, BIRC5, CDK1, ASF1B, E2F1, RACGAP1, MYBL2, TPX2, CDC20, TOP2A, NUSAP1, UBE2T, LMNB1, CCNB1, ZWINT, STMN1, and TK1). A majority of them were demonstrated to exert essential roles in the pathogenesis of PCa [25–27]. Among these hub genes, we chose four seldomly reported in PCa, namely TK1, RACGAP1, ZWINT, and LMNB1, to explore their diagnostic and prognostic value. Thymidine kinase 1 (TK1), a cell cycle-dependent protein, has been extensively reported as a tumor biomarker [28], but its role in PCa remained unclear. RACGAP1 encodes a GTPase-activating protein (GAP) that plays a regulatory role in cytokinesis, cell growth, and differentiation. RACGAP1 has been widely reported to take part in the pathogenesis of various cancers, such as colorectal cancer [29], hepatocellular carcinoma [30], and others, but its role in the development of PCa was also unclear. Similarly, the diagnostic and prognostic roles of ZWINT and LMNB1 in PCa remained so far obscure. We determined that TK1, RACGAP1, ZWINT, and LMNB1 were not only significantly up-regulated in PCa tissues, but correlated positively as well with higher Gleason score and TNM stage, suggesting important contributions to the pathogenesis of PCa. Furthermore, ROC curves showed that all four genes could serve as biomarkers to distinguish tumors from normal prostate tissue sensitively and accurately. Indeed, all these genes appeared as promising candidates as therapeutic targets and prognosis predictors.

We also referred to DiseaseMeth 2.0 and MEXPRESS to explore DNA methylation patterns that could account for the abnormal expression of the above hub genes in PCa. We found that TK1, RACGAP1, ZWINT, and LMNB1 were hypomethylated in PCa samples compared with adjacent normal ones, which is consistent with the observed up-regulation of these four hub genes in PCa.

To further explore hub genes’ biological functions, we referred to TIMER and conducted GSEA and GSVA for each hub gene. The expression of LMNB1, TK1, ZWINT, and RACGAP1 was positively associated with tumor purity, while no or weak associations were observed for hub genes and infiltrating immune cells in PCa tissue samples. Based on the findings from TIMER, we proposed that LMNB1, TK1, ZWINT, and RACGAP1 are mainly expressed in PCa cells rather than immune cells, and their functions do not relate to immunological regulation of the tumor microenvironment. The results of GSEA and GSVA were in accordance with this speculation. Also, many cell cycle-related KEGG pathways, such as homologous recombination, mismatch repair, and DNA replication, were enriched in the high-expression groups of these hub genes, suggesting their contribution to PCa proliferation.

In conclusion, by combining RRA, WGCNA, and other bioinformatics tools we identified and characterized several robust DEGs and significant gene modules in PCa. Four hub genes (RACGAP1, ZWINT, TK1, and LMNB1) were strongly upregulated in PCa tissues, an expression pattern likely associated with hypomethylation. GSEA and GSVA further suggested that these genes highly influenced the development of PCa. More work needs to be done to fully reveal their contribution to the pathogenesis of PCa, and to validate their usefulness as diagnostic and/or prognostic markers.

## MATERIALS AND METHODS

### Selection of PCa gene expression datasets

All eligible microarray datasets were downloaded from GEO. The selection criteria were as follows: 1) Inclusion of gene expression data of PCa and adjacent normal tissue samples; normal prostate tissue samples from donors and benign prostatic hyperplasia tissue samples were excluded; 2) Arrays contained a minimum of 10 tumor and adjacent normal tissue samples; 3) Inclusion of >5,000 genes in the GEO platform. According to the above screening criteria, 10 datasets were finally included in this study: GSE6919 [31], GSE6956 [32], GSE32448 [33], GSE32571 [34], GSE35988 [35], GSE46602 [36], GSE68555 [37], GSE69223 [38], GSE70768 [39], and GSE88808 [40] (Table 1). In addition, PCa RNA-sequencing and clinical data were downloaded from the TCGA database (https://cancergenome.nih.gov/) and utilized in the study.

### Identification of robust DEGs

We downloaded the series matrix files of datasets from GEO. The R package “limma” [41] was utilized to normalize the data and find DEGs. We then used RRA to integrate the results of those 10 datasets to find the most significant DEGs [11]. The P value of each gene indicated its ranking in the final gene list, and genes with adjusted P < 0.05 were considered as significant DEGs in the RRA analysis.

### Visualization of gene expression patterns and chromosome locations

“OmicCircos” (R package) was utilized to visualize the expression patterns and chromosomal locations of the top 100 DEGs (top 50 up-regulated genes and top 50 down-regulated genes according to adjusted P) from RRA analysis.

### Function enrichment analyses

We conducted Gene Ontology (GO) enrichment and Kyoto Encyclopedia of Genes and Genomes (KEGG) pathway analyses using the R package “clusterprofiler” [42]. GO terms or KEGG pathways with adjusted P < 0.05 were considered statistically significant and visualized by “GOplot” (R package) [43].

### WGCNA

We extracted the top 5,000 up-regulated DEGs (according to P) from RRA analysis to perform WGCNA with expression data retrieved from TCGA. The R package “WGCNA” was applied to find clinical traits-related modules and hub genes among them [44]. The adjacency matrix was transformed into topological overlap matrix (TOM). According to the TOM-based dissimilarity measure, genes were divided into different gene modules. Here, we set soft-thresholding power as 6 (scale free R^2^ = 0.85), cut height as 0.25, and minimal module size as 10 to identify key modules. The module with the highest correlation with clinical traits was selected to explore its biological function through GO and KEGG analyses and to screen hub genes. Hub genes were defined as those with gene significance (GS) > 0.3 and module membership (MM) > 0.8.

### Validation and survival analysis of hub genes

We utilized “ggstatsplot” (R package, https://cran.r-project.org/web/packages/ggstatsplot/) to validate the hub genes’ expression levels between PCa and adjacent normal tissue samples as well as their correlations with clinical features in The Cancer Genome Atlas Prostate Adenocarcinoma (TCGA-PRAD) dataset. Independent samples T test or one-way analysis of variance (ANOVA) was used as appropriate. To assess hub genes’ diagnostic values, we plotted receiver operating characteristic (ROC) curves and calculated area under the ROC curve (AUC) with “pROC” R package [45]. Survival analysis was also conducted for hub genes using “survminer” (R package, https://CRAN.R-project.org/package=survminer) and “survival” (R package, https://CRAN.R-project.org/package=survival). Tumor samples within the TCGA-PRAD dataset were divided into two groups based on each hub gene’s best-separation cut-off value to plot the Kaplan-Meier (K-M) survival curves.

### Methylation and gene expression analyses

The human disease methylation database version 2.0 (DiseaseMeth 2.0, http://bioinfo.hrbmu.edu.cn/diseasemeth/) incorporates methylome data from microarray and sequencing technology and annotates DNA methylation status in human diseases [46, 47]. We utilized this website to compare methylation levels of hub genes between the PCa and paracancerous normal tissues. Furthermore, we investigated the association between hub genes’ expression and their DNA methylation status using MEXPRESS (http://mexpress.be) [48], a web tool for integrating and visualizing clinical data from TCGA, gene expression, and DNA methylation.

### Analysis of gene expression and tumor-infiltrating immune cells

To investigate the correlation between the expression of selected hub genes and tumor infiltrating immune cells (B cells, CD4^+^ T cells, CD8^+^ T cells, neutrophils, macrophages, and dendritic cells), we applied the online tool TIMER (https://cistrome.shinyapps.io/timer/) [49, 50] which contains 10,897 samples from diverse cancer types available in the TCGA database.

### Gene Set Enrichment Analysis (GSEA) and Gene Set Variation Analysis (GSVA)

We utilized the R package “clusterprofiler” [42] to perform GSEA analysis of hub genes with TCGA-PRAD RNA-seq data. In addition, the “GSVA” R package was used to find the pathways most associated with hub genes [51]. Based on the median expression of each hub gene, 498 PCa samples were divided into two groups (high expression vs low expression). P < 0.01 was regarded as statistically significant. The gene set “c2.cp.kegg.v6.2.symbols.gmt”, downloaded from the Molecular Signature Database (MSigDB, http://software.broadinstitute.org/gsea/msigdb/index.jsp), was selected as the reference gene set.

## Supplementary Material

Supplementary Figures
