# Supplementary material for: Identification of hub genes in prostate cancer using robust rank aggregation and weighted gene co-expression network analysis
Source: Aging (Albany NY). 2019 Jul 15;11(13):4736–56. doi: 10.18632/aging.102087 (PMC6660050; doi:10.18632/aging.102087)
Supplement: Supplementary Figures [file aging-11-102087-s001.pdf]

SUPPLEMENTARY FIGURES

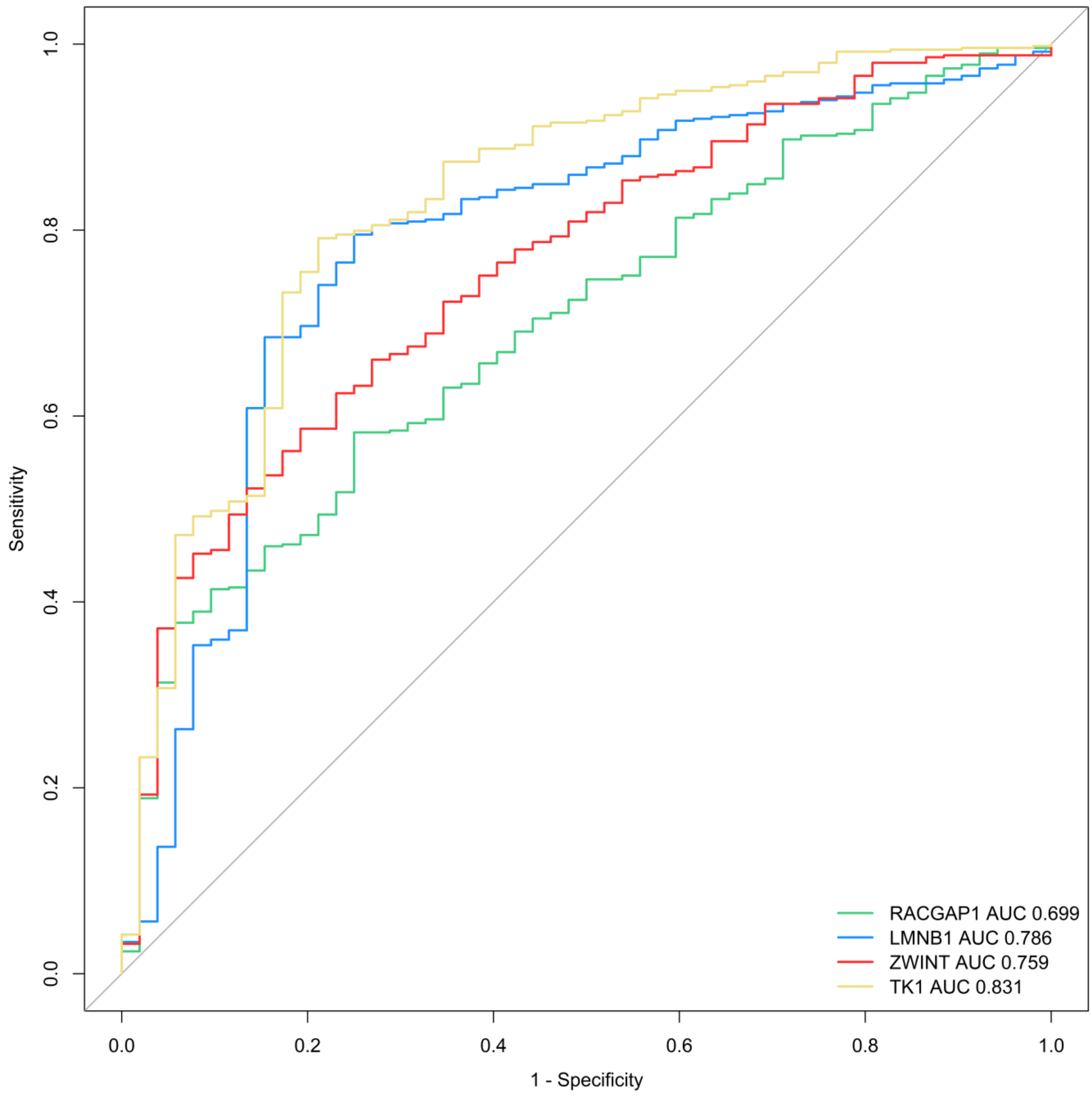

Supplementary Figure 1. ROC curves for LMNB1, TK1, RACGAP1, and ZWINT. ROC, receiver operating characteristic; AUC, area under the ROC curve.

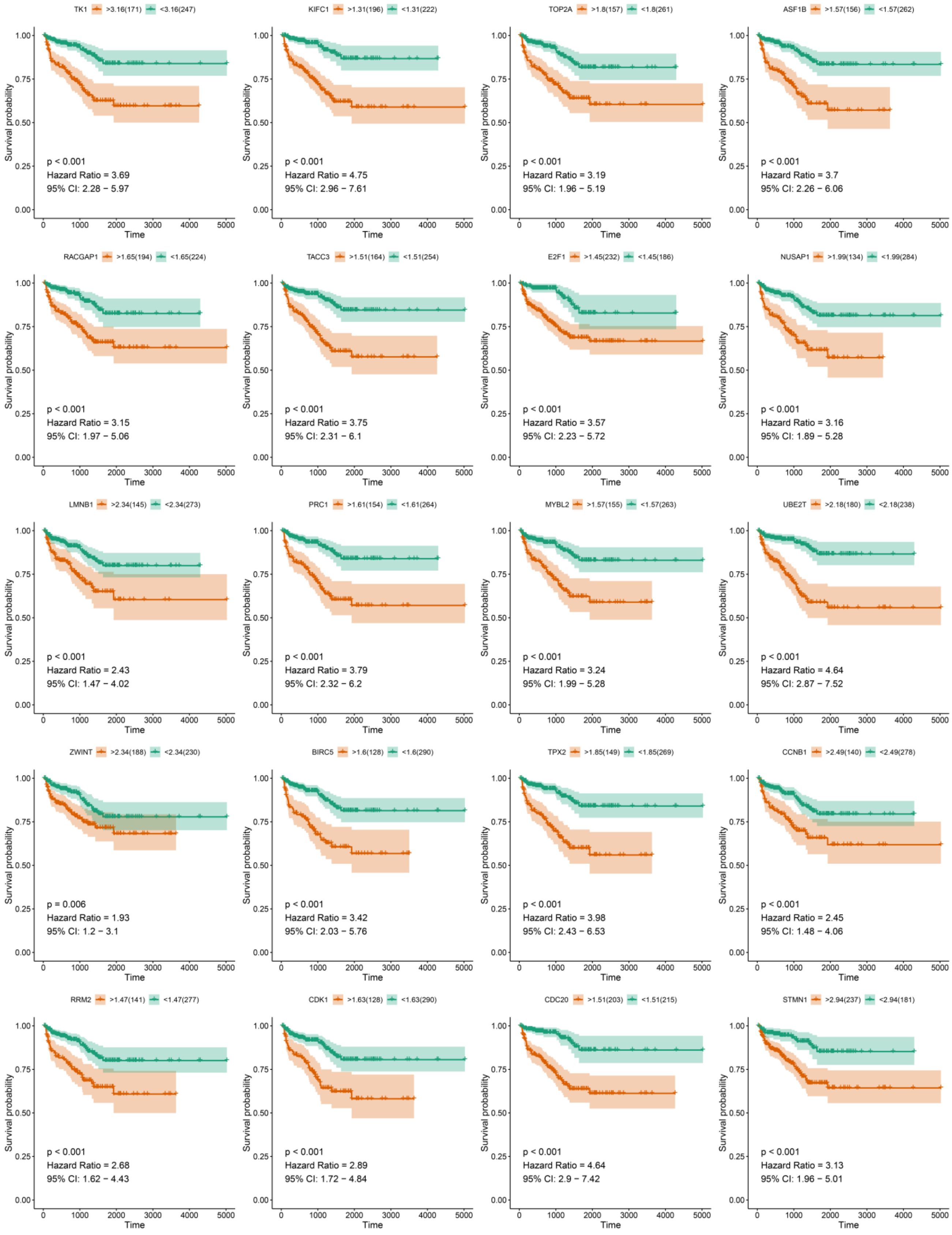

Supplementary Figure 2. Survival analysis of all hub genes in the WGCNA pink module. Kaplan–Meier plots of disease-free survival in two groups divided by each hub genes' best-separation value.

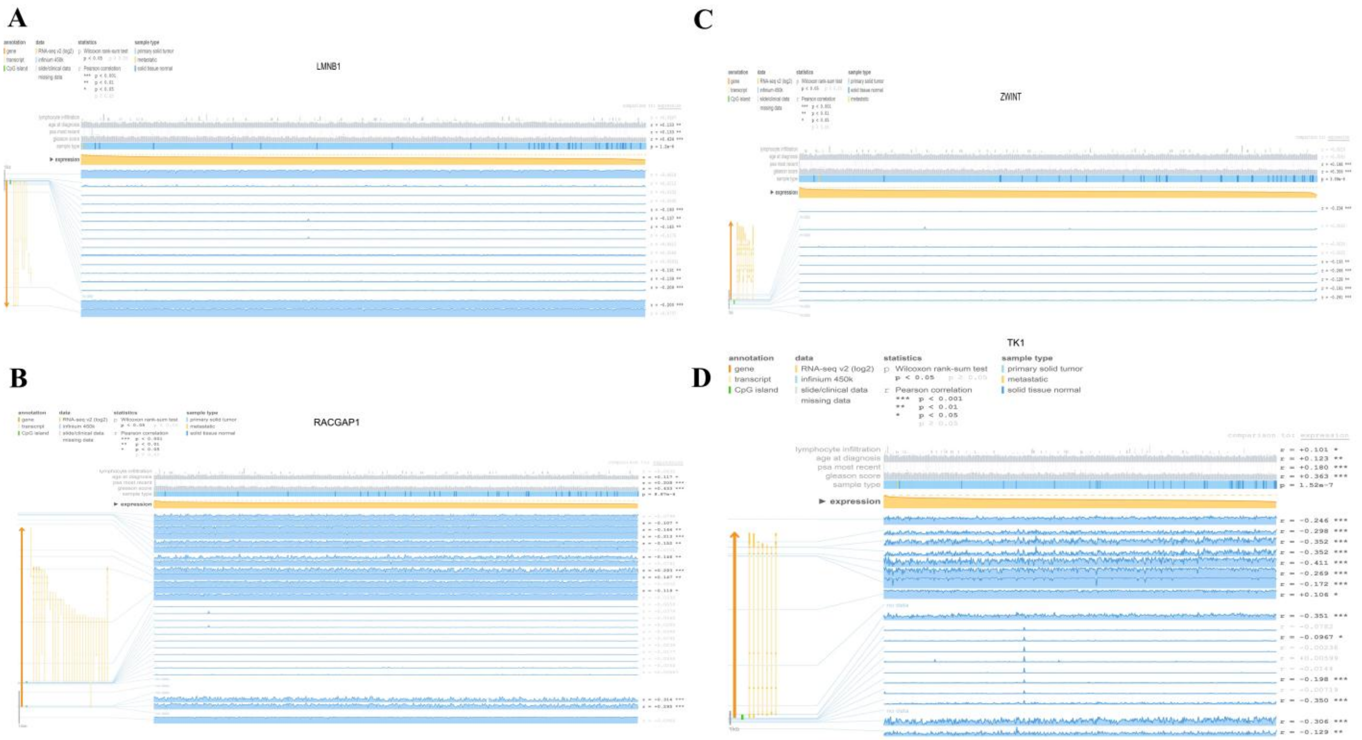

Supplementary Figure 3. Association of Methylation sites with expression of PCa hub genes. The methylation sites of (**A**) LMNB1, (**B**) RACGAP1, (**C**) ZWINT, and (**D**) TK1 DNA sequences, and their associations with gene expression, were visualized using MEXPRESS. The expression of query genes is illustrated by the orange line in the center of the plot. The samples are ordered by query gene expression (normalized RNASeqV2 values in TCGA). Pearson's correlation coefficients and P values from Wilcoxon rank-sum test for methylation sites and query gene expression are shown on the right side. The blue lines stand for Infinium 450k probes and their heights represent the beta value for this probe. Dark yellow and green lines at the bottom left indicate the query gene and CpG islands.
